# Quantification of Massive Seasonal Aggregations of Blacktip Sharks (*Carcharhinus limbatus*) in Southeast Florida

**DOI:** 10.1371/journal.pone.0150911

**Published:** 2016-03-30

**Authors:** Stephen M. Kajiura, Shari L. Tellman

**Affiliations:** Department of Biological Sciences, Florida Atlantic University, Boca Raton, Florida, United States of America; Department of Agriculture and Water Resources, AUSTRALIA

## Abstract

Southeast Florida witnesses an enormous seasonal influx of upper trophic level marine predators each year as massive aggregations of migrating blacktip sharks (*Carcharhinus limbatus*) overwinter in nearshore waters. The narrow shelf and close proximity of the Gulf Stream current to the Palm Beach County shoreline drive tens of thousands of sharks to the shallow, coastal environment. This natural bottleneck provides a unique opportunity to estimate relative abundance. Over a four year period from 2011–2014, an aerial survey was flown approximately biweekly along the length of Palm Beach County. A high definition video camera and digital still camera mounted out of the airplane window provided a continuous record of the belt transect which extended 200 m seaward from the shoreline between Boca Raton Inlet and Jupiter Inlet. The number of sharks within the survey transect was directly counted from the video. Shark abundance peaked in the winter (January-March) with a maximum in 2011 of 12,128 individuals counted within the 75.6 km^-2^ belt transect. This resulted in a maximum density of 803.2 sharks km^-2^. By the late spring (April-May), shark abundance had sharply declined to 1.1% of its peak, where it remained until spiking again in January of the following year. Shark abundance was inversely correlated with water temperature and large numbers of sharks were found only when water temperatures were less than 25°C. Shark abundance was also correlated with day of the year but not with barometric pressure. Although shark abundance was not correlated with photoperiod, the departure of the sharks from southeast Florida occurred around the vernal equinox. The shark migration along the United States eastern seaboard corresponds spatially and temporally with the spawning aggregations of various baitfish species. These baseline abundance data can be compared to future studies to determine if shark population size is changing and if sharks are restricting their southward migration as global water temperatures increase.

## Introduction

The blacktip shark (*Carcharhinus limbatus* Müller & Henle, 1839) is a cosmopolitan species in tropical and warm temperate waters around the world [1]. This species is found on or adjacent to continental or insular shelves, as well as far offshore, but it is not truly oceanic [1]. In the eastern United States the blacktip ranges from New England to the Florida Keys, and the Gulf of Mexico [2]. It is found primarily south of Cape Hatteras, North Carolina, occurring north of there only as “a rare stray” [3]. The blacktip is a medium sized shark with a maximum length of approximately 2 m in the northwestern Atlantic and Gulf of Mexico [2]. This species feeds primarily upon teleosts, along with small elasmobranchs, cephalopods and crustaceans [1].

The blacktip shark is harvested in commercial and artisanal fisheries worldwide [4–11] but receives sophisticated management only in Australia and the United States [12]. In the United States the blacktip shark is the primary target of a directed commercial fishery along the southeast coast and in the Gulf of Mexico [13]. Florida has the largest commercial shark fishery of any southeastern state, although commercial landings in Florida represent sharks harvested from federal, not state, waters [14]. In Florida the blacktip shark is caught primarily with commercial longline gear but localized gillnet fisheries will target blacktips during their migrations [15]. The blacktip is second only to the sandbar shark (*Carcharhinus plumbeus*) in commercial landings in Florida [14]. Both species are harvested for their meat and the meat of the blacktip is considered to be superior to the sandbar shark [12, 15]. In addition to commercial harvest, the blacktip shark is fished recreationally in the United States, particularly in Florida, although the recreational CPUE has declined since the 1980s [15].

**Fig 1:**
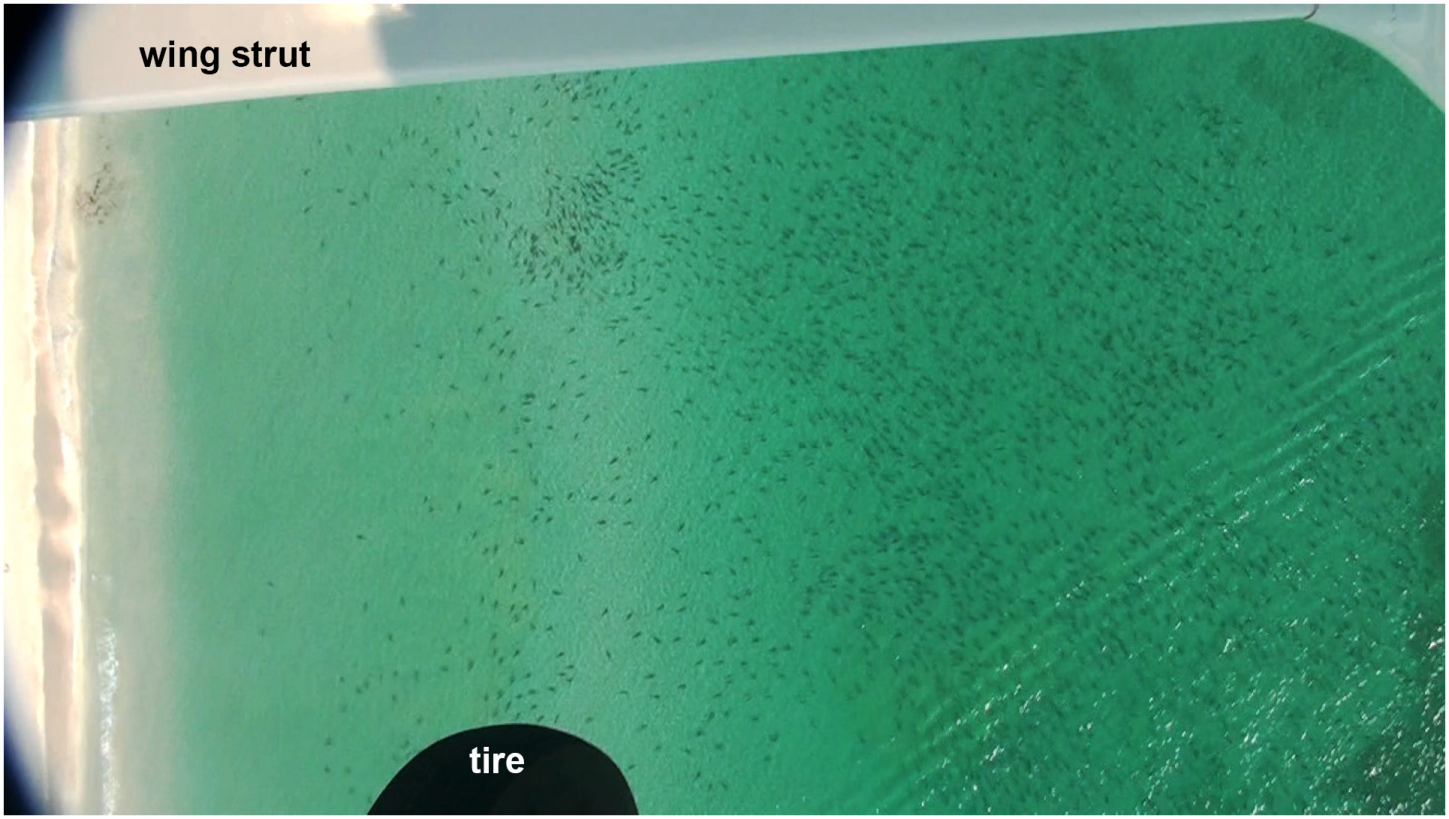
Sample frame from high definition video. This single frame illustrates a large shark aggregation in the nearshore environment. The lateral field of view is approximately 200 m and there are approximately 1,678 sharks visible.

Each winter in southeast Florida, thousands of blacktip sharks form large aggregations in the shallow water immediately off shore (Fig 1). The clear water and light colored, sandy seafloor allow the sharks to be easily visualized from the air and the enormous numbers of sharks in such close proximity to popular swimming beaches always captures local and national media attention. Beaches are often closed due to the presence of sharks. However, despite the large numbers of sharks, bites on humans are relatively infrequent [1]. These large seasonal aggregations are thought to represent the overwintering phase of the blacktip shark’s annual migration along the United States eastern seaboard [2].

**Fig 2:**
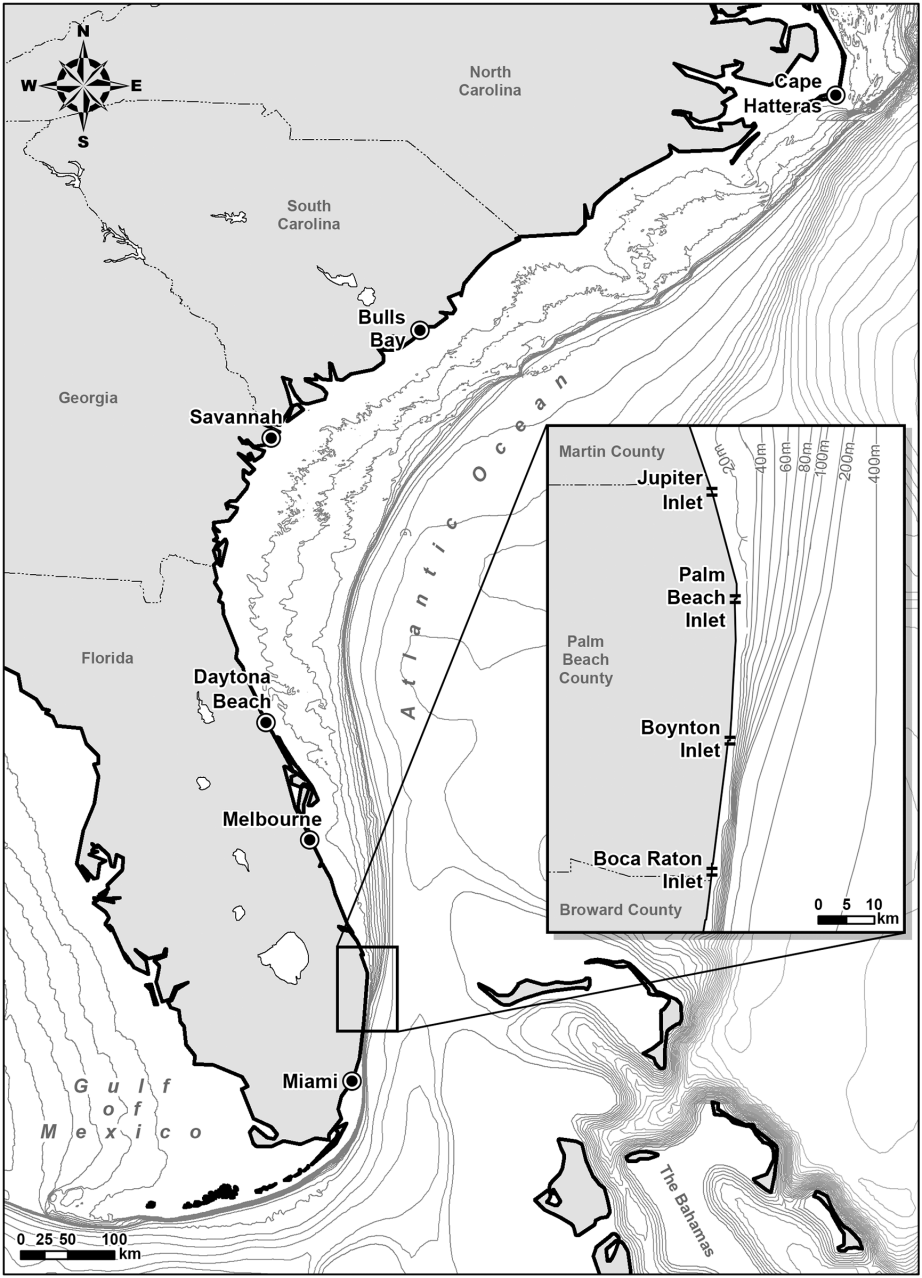
Bathymetric map. Bathymetric map of the blacktip shark distribution range along the United States eastern seaboard. The broad shelf narrows dramatically in Palm Beach County, Florida (inset). Aerial survey flights were conducted between Boca Raton Inlet and Jupiter Inlet.

This annual migration in the western Atlantic has been previously constructed from catch data [2, 16]. The blacktips overwinter in southeast Florida and begin their northward migration in March [2]. They are caught in large numbers off Daytona Beach, Florida in March and April [16] (Fig 2). Farther north, blacktips are abundant in Bulls Bay, South Carolina from May to September [16]. By May to June, the migration has reached North Carolina [2] and blacktips are rarely reported farther north [3]. However, tagging data indicate that they can be found as far north as Delaware Bay [17]. The sharks spend summer in the coastal waters off North Carolina, South Carolina, and Georgia before beginning their southward migration in September to October [2]. The blacktips again peak in abundance off Daytona Beach, Florida in September through November [16] and off Melbourne Beach, Florida in November and December [18].

The blacktip shark migration along the United States eastern seaboard has been previously associated with changing water temperature. The sharks begin their southward migration from Bulls Bay, South Carolina in October when the water temperature drops below 21°C [16]. Similarly, on their northward migration, they are found off Daytona Beach, Florida in March and April when the water temperature is 18–21°C [16]. Although temperature is the obvious correlate with the shark migration, other environmental factors may also contribute to the initiation of movement [19]. In other elasmobranch species, seasonal changes in photoperiod serve as a driver to initiate movement [20–22]. Biotic factors, such as seasonal prey availability, may also contribute to the movement of elasmobranch predators [23–25]. Because temperature, photoperiod, and prey availability often co-vary, it is difficult to ascertain which factor is primarily responsible for driving the migration.

Although this annual migration is well known, there remains a dearth of empirical data on blacktip shark abundance as no rigorous studies of this phenomenon have been conducted. Southeast Florida is the presumed overwintering grounds for the blacktips [2], but this presumption is only based on anecdotal evidence, and the species’ seasonal abundance and associated environmental parameters have not been quantified. Therefore, the goal of this study was to quantify shark abundance on a seasonal basis and correlate the presence of these massive aggregations with various environmental factors, including water temperature, the presumed driving factor for their movement. To accomplish this we employed an aerial survey technique which capitalized upon the clear water and close proximity of the sharks to the shore.

## Materials and Methods

Aerial survey flights were conducted approximately biweekly from 04 February 2011 through 17 April 2013, and from 04 January through 01 April 2014. The survey transect extended from Boca Raton Inlet (26° 20’ 09" N, -80° 4’ 16" W) northward along the shoreline to Jupiter Inlet (26° 56’ 38" N, -80° 4’ 16" W), a distance of 75.6 km (Fig 2). Flights were flown at an altitude of approximately 150 m and an airspeed of approximately 150 km h^-1^. This combination of altitude and airspeed provided sufficient resolution to easily distinguish individual animals. Survey flights were conducted only on days in which the wind speed and direction produced relatively calm sea surface conditions which facilitated viewing into the water. Flights were flown in the mornings between 0800–1100 local time, which provided optimal lighting with minimal surface glare. During each flight water clarity was ranked from 1 (excellent) to 5 (poor). To provide consistency in the evaluation of water clarity, the authors flew most of the flights together and came to a consensus on the clarity rank for each flight. At least one of the authors was present on every flight. Water temperature and barometric pressure data were acquired for each day at 1000 local time, approximately in the middle of the survey flight time. The data were acquired from the National Data Buoy Center for the Lake Worth Pier Station (http://www.ndbc.noaa.gov/station_history.php?station=lkwf1) which is located at approximately the midpoint of the survey transect. Photoperiod data for Lake Worth, Florida were collected from the United States Naval Observatory (aa.usno.navy.mil/data/docs/RS_OneYear.php).

**Fig 3:**
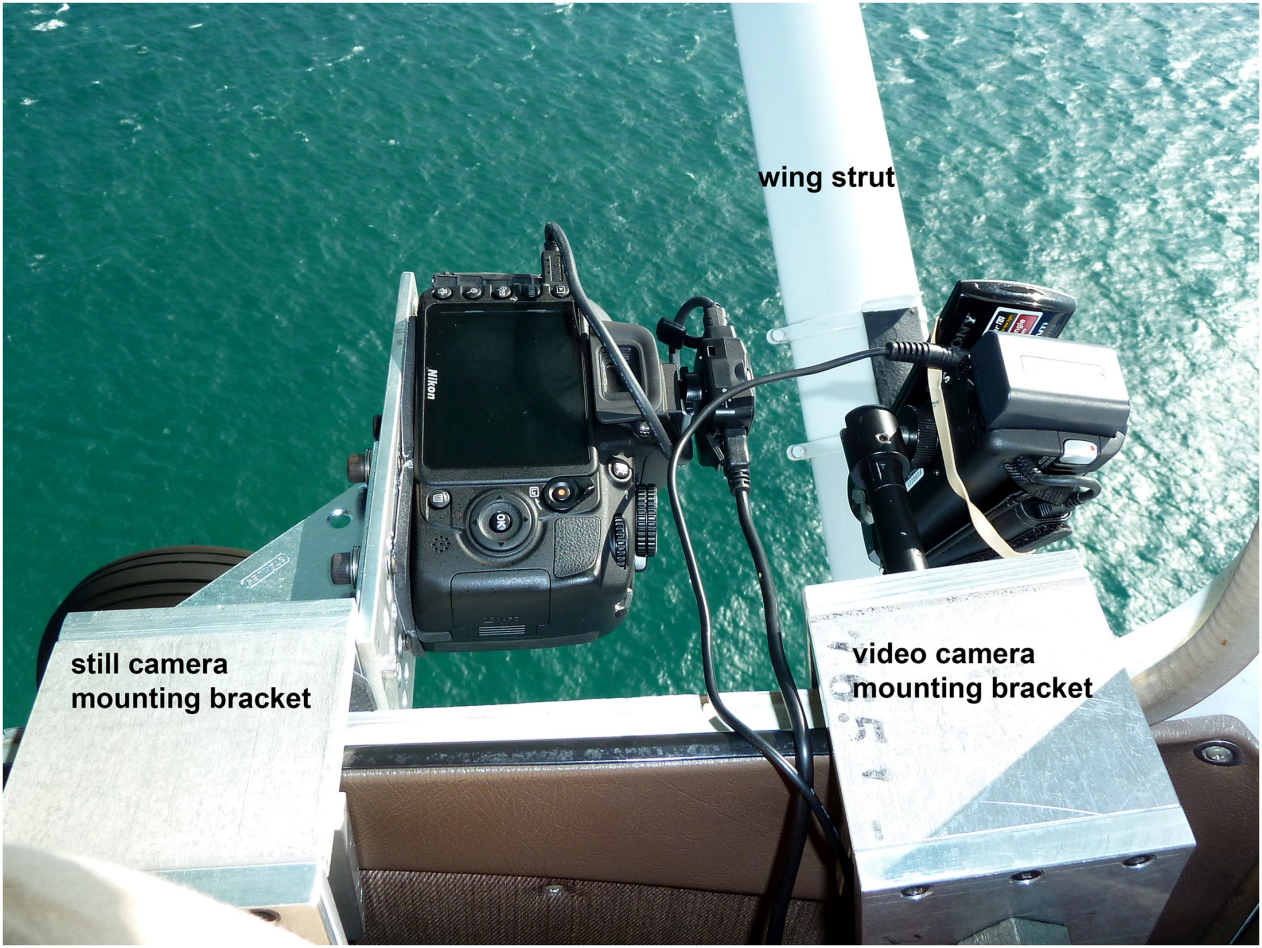
Experimental setup for recording aerial surveys. A high definition video camera and digital SLR camera are mounted on custom brackets out of the open window of the aircraft.

To quantify shark abundance, a high definition (1080p) video camera with GPS capabilities (Sony HDR-CX160) was mounted on a custom fabricated bracket out of the open pilot’s side window of a Cessna 172 aircraft (Fig 3). A cable from the cockpit audio panel to the camera enabled recording of all cockpit communications. The camera was outfitted with a circular polarizing filter to reduce sea surface glare, and was positioned with the lens aimed straight downward. The plane flew northward parallel to the shore and at a distance approximately 200 m offshore. This enabled the video camera to record a belt transect with a lateral field of view from the shoreline to approximately 200 m offshore during flight. The field of view was determined by georeferencing to the Lake Worth pier, which extends 265 m from the shoreline. The total area surveyed along the 75.6 km transect was approximately 15.1 km^-2^ and sharks outside of the belt transect were not counted.

Starting on June 22 2011, a 35mm digital SLR camera (Nikon D3100) was mounted on a custom fabricated bracket immediately behind the video camera and also positioned looking straight downward. The SLR camera was outfitted with a circular polarizing filter and had a GPS unit (Nikon GP-1A) attached which recorded location information for each frame. An intervalerometer was programmed to record a single still frame every two seconds which provided overlap between successive frames. These still photos served as a backup to the video footage and provided a higher resolution image (14.2 megapixels) to facilitate counting of dense aggregations, if necessary. The lateral field of view for the two cameras was nearly identical.

Video footage and still frames were downloaded in the laboratory using iMovie (v8.0.6) and iPhoto (v8.1.2) software, respectively. The video footage was carefully reviewed and correlated with comments on the audio track that noted the presence of sharks. For footage in which few sharks were visible, the number of sharks was tallied directly from the video. When large numbers of sharks were present, individual frames were extracted from the video, imported into ImageJ (v1.43), and two independent reviewers manually counted the number of sharks in each frame. For large aggregations that spanned across successive frames, care was taken to avoid overlapping the field of view of subsequent frames and consequently over-counting the number of sharks. The number of sharks was collated for the entire survey flight for both reviewers and the mean number is reported. All sharks were assumed to be *C*. *limbatus*, unless obviously another species.

For dates when water clarity ranked as 5 (poor) the shark abundance data were considered unreliable and were excluded from analysis. To test for seasonal differences in shark abundance, a Kruskal-Wallis test was applied. Because of the high degree of temporal repeatability, shark abundance data were pooled by quarter (January-March; April-June; July-September; October-December) over all four years and abundance was compared among quarters. To determine which environmental variables correlated with shark abundance, a Generalized Linear Model was applied with day of the year, water temperature (°C), photoperiod (minutes), and barometric pressure (hPa) as the predictor variables. Because of the time dependent nature of the model, data were analyzed only for the period of continuous sampling (February 2011—April 2013) and did not include the shark abundance data from January-April 2014. The models were evaluated for the lowest AIC and highest adjusted coefficient of determination (R^2^). A variance inflation factor (VIF) was calculated to determine multicollinearity for each predictor in the model. A Spearman’s Rank Correlation was subsequently applied to determine the relationships between shark abundance and the four predictor variables (day of the year, water temperature, photoperiod, and barometric pressure).

## Results

A total of 58 survey flights were conducted with a total of 104,255 sharks counted within the belt transect (S1 Table). Sharks were found singly, in small groups, or large aggregations up to thousands of individuals. When found in small groups the sharks were typically swimming in a polarized school. However, in large aggregations the sharks did not necessarily swim in a polarized school, and individuals were often oriented in different directions (Fig 1). Although the sharks were not directly observed feeding, they were sometimes seen in close proximity to schools of baitfish. Sharks were also seen to jump out of the water, but it was difficult to determine whether they exhibited any other social behaviors.

**Fig 4:**
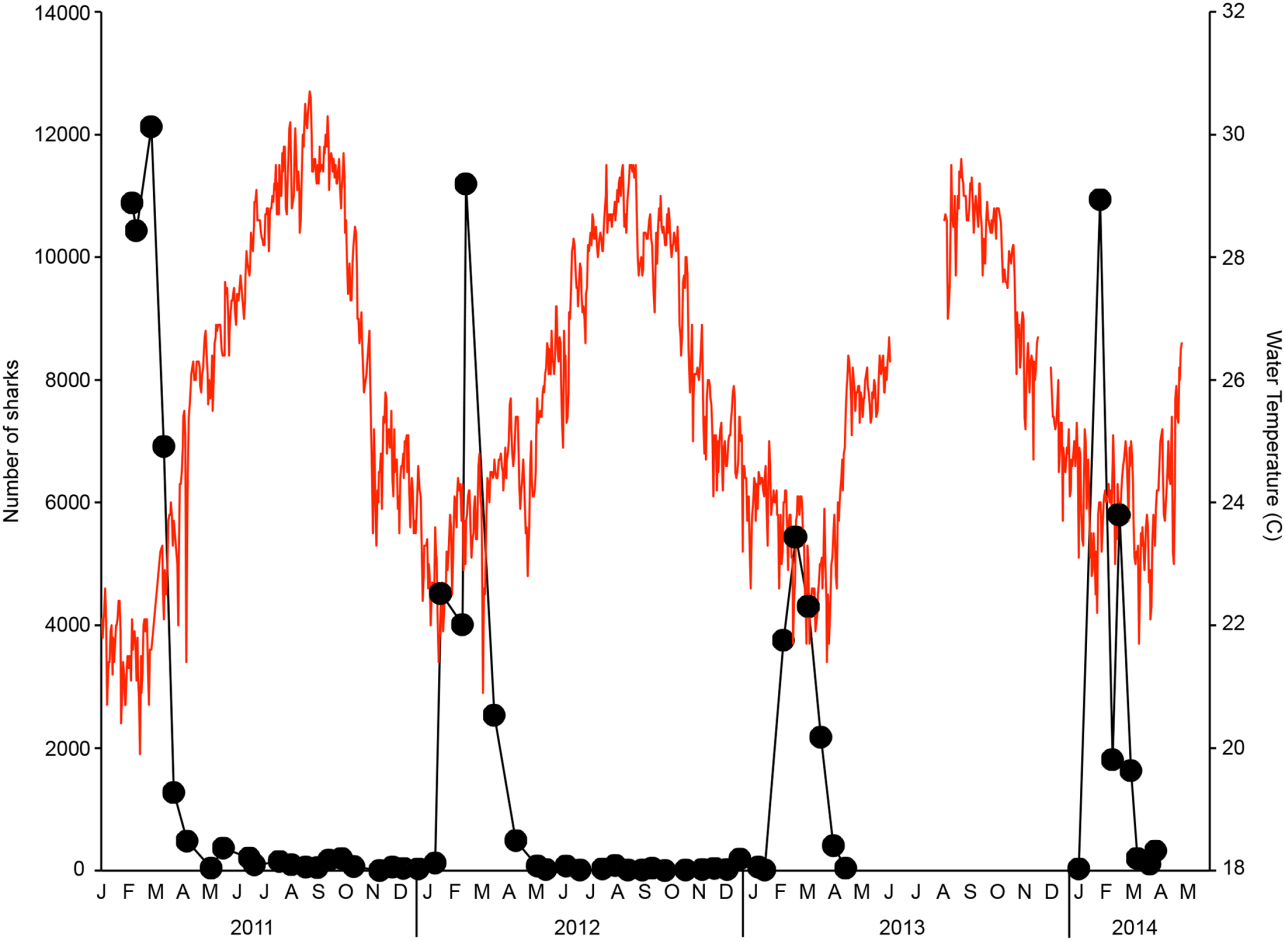
Seasonal shark abundance and water temperature. The number of sharks (black circles) correlates inversely with water temperature (red lines). Shark abundance peaks from January-March which corresponds with the lowest water temperatures.

The number of sharks counted per survey varied with season (Fig 4). Shark abundance was greatest in the winter months (January-March) with a peak winter seasonal abundance within the belt transect of 9925.0 individuals averaged over all years. Shark abundance declined precipitously in the spring and very few sharks were seen in the surveys during the summer and fall months (May-December). Summer and fall shark abundance averaged 111.7 individuals over all years, approximately 1.1% of the winter peak. Mean shark abundance differed among quarters (Kruskal-Wallis, χ^2^ = 24.640, df = 3, p<0.0001). Post-hoc analysis revealed that mean shark abundance for the first quarter (January-March) was significantly greater than for the other three quarters (April-June, χ^2^ = 10.447, p = 0.001; July-September, χ^2^ = 13.831, p<0.0001; October-December, χ^2^ = 11.772, p = 0.001). Shark abundance in the second quarter (April-June) was also significantly greater than in the fourth quarter (October-December, χ^2^ = 5.010, p = 0.025).

Shark abundance within the transect area reached a peak of 12,128 individuals in February 2011. This resulted in a peak density of approximately 803.2 sharks km^-2^. In contrast, lowest shark density occurred in the third and fourth quarters (July-September, October-December) yielding an average density of approximately 4.1 sharks km^-2^. Sharks were distributed from just a few meters from the shore throughout the entire field of view (S1 Movie) and could also be seen on the seaward side of the plane, although those individuals were outside the field of view of the cameras and were not counted. The maximum depth of the water throughout the survey transect was <4 m and it was possible to visualize details on the seafloor throughout the entire transect area. This provided confidence that all sharks within the belt transect were visible, and not obscured by water depth. Sharks were found throughout the entire survey transect but generally in greater numbers from Boynton Inlet to Jupiter Inlet. All sharks were approximately the same size and blacktips sampled from the large winter aggregations averaged 173.4 cm total length (n = 35) (Kajiura, unpublished data).

The Generalized Linear Model predicting shark abundance by four variables (day of the year, water temperature, photoperiod, barometric pressure) was significant (F = 17.88, df = 2, p<0.0001). Upon comparing AIC criteria and adjusted R^2^, the best model was achieved with the parameters water temperature and day of the year (adjusted R^2^ = 0.4399, AIC = 692.3353). The individual parameter estimates were significant for both water temperature (p = 0.0007) and day of the year (p = 0.0332). The variance inflation factor (VIF) was equal to 1.341, which indicates that multicollinearity among temperature and day of the year is likely not a confounding factor.

**Fig 5:**
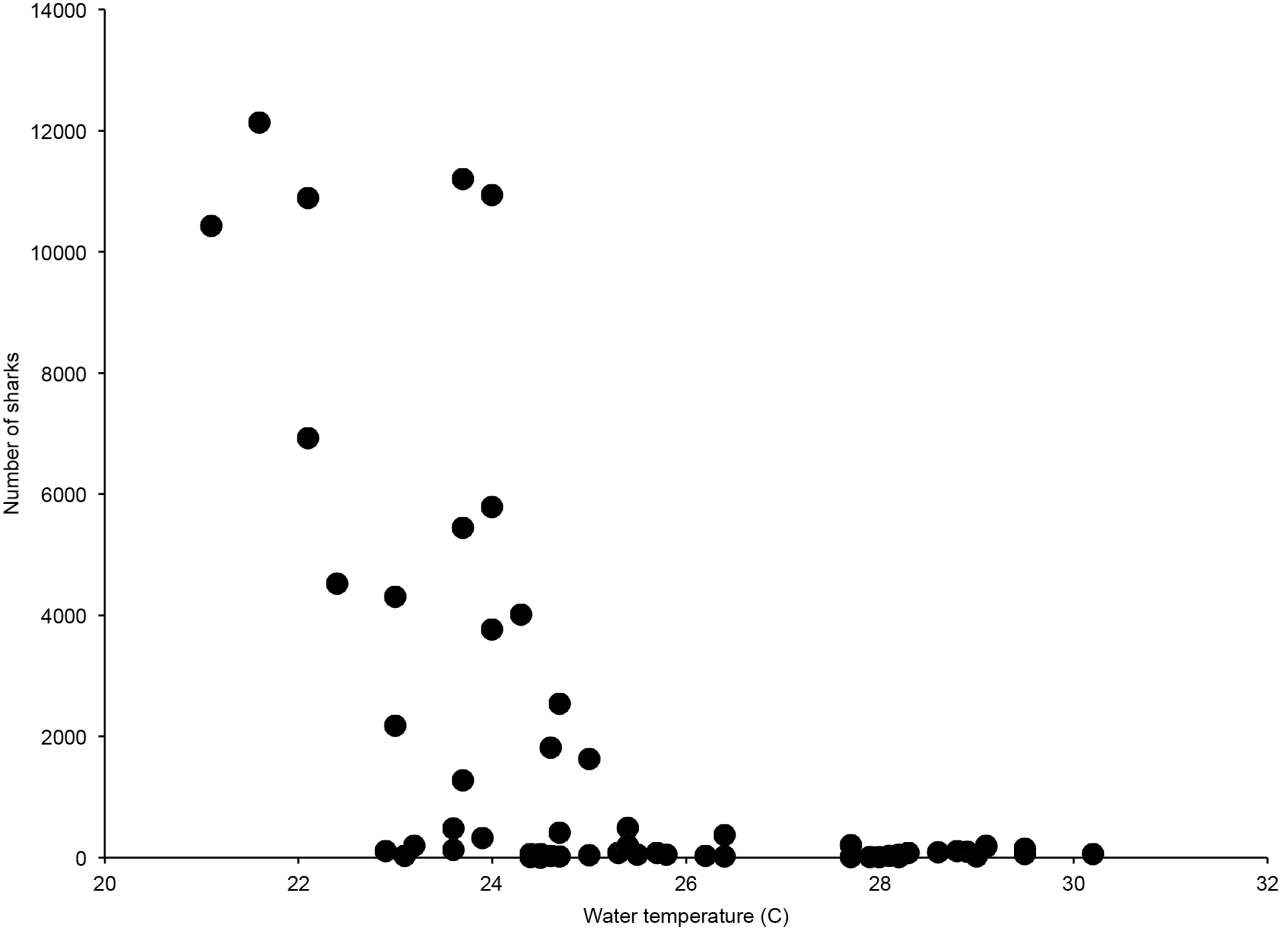
Shark abundance vs water temperature. Large numbers of sharks are found only at water temperatures of less than 25°C.

Shark abundance was inversely correlated with both water temperature and day of the year (Spearman’s rank correlation, water temperature: ρ = -0.581, p<0.0001; day of the year: ρ = -0.594, p<0.0001) (Fig 4). Sharks were present in greatest numbers when water temperature was less than 25°C (Fig 5). In contrast, photoperiod did not show a significant correlation with shark abundance (Spearman’s rank correlation, ρ = -0.137, p = 0.320). However, the seasonal decline in shark abundance corresponded closely with the vernal equinox.

## Discussion

This study provides the first quantitative assessment of blacktip shark abundance in their winter aggregation site off Palm Beach County, Florida. Although their migratory movements have been previously reconstructed from catch records [2, 16], this study employs high temporal resolution sampling, along a set belt transect, over multiple years, to quantify shark abundance at a single point along their migratory route. It is only possible to assess the number of individuals involved because the blacktip sharks aggregate in shallow water close to shore where they can be easily seen and counted.

### Spatial distribution drivers

The spatial distribution of blacktip sharks very close to shore in southeast Florida is likely attributable to both biotic and abiotic factors. Blacktip sharks are typically associated with continental and insular shelves [1]. In the northern part of their range, from the Carolinas to central Florida, the shelf extends far from shore and the sharks have the potential to be widely distributed seaward (Fig 2). The shelf narrows dramatically in Palm Beach County, Florida, and southward migrating sharks would necessarily be funneled in close to shore. The Gulf Stream current originates at the southern tip of Florida and flows northward along the United States eastern seaboard, closely following the continental shelf, from the Florida Straits to Cape Hatteras before being deflected eastward out to sea [26]. Off southeast Florida, the Gulf Stream averages about 80 km in width, extends to a depth of 800 m, and has a maximum surface velocity of approximately 2.5 m s^-1^ [27]. Southward migrating sharks could minimize the energetic cost of their migration by remaining close to the shore and away from this large, strong, northward flowing current. The bathymetry and hydrology jointly contribute to the sharks being driven to the nearshore environment.

In addition to abiotic factors, the baitfish upon which the sharks are presumed to feed are largely distributed close to shore [16]. Although we did not directly observe predation we did see sharks in close proximity to large schools of baitfish. Similarly, the blacktip is prey to larger predatory sharks such as the tiger shark (*Galeocerdo cuvier*) and great hammerhead (*Sphyrna mokarran*) [2]. The presence of these larger predators might exert pressure for the blacktips to refuge in the shallow, nearshore environment, as documented for various other shark species [28, 29]. The blacktips might also be utilizing the shallow nearshore waters for thermoregulation [19]. Because their seasonal movement is strongly correlated with temperature, it is plausible that these sharks are sensitive to small temperature changes. This could result in microhabitat selection for their preferred temperature range in warmer nearshore waters compared to the cooler water found offshore below the thermocline. The warmer nearshore water might augment metabolic and physiological functions including digestion and somatic growth [30–32]. Therefore, the large aggregations might form as a function of feeding, predator avoidance, thermoregulation, or any combination.

### Temporal movement pattern

The blacktip sharks exhibit a defined movement pattern along the United States eastern seaboard, as previously described [2, 16]. Along the east coast of central Florida (Melbourne Beach and Daytona Beach) there are two seasonal peaks in blacktip shark abundance—one in the spring and one in the fall [16, 18]. The two peaks suggest that the sharks are transiting through those areas as part of their northward and southward migration. In contrast, the single annual peak in abundance off Palm Beach County suggests that southeast Florida is likely the southernmost terminus of their migration.

The blacktip shark movement pattern is closely correlated with water temperature and prey abundance. Various baitfish species included in the blacktip shark diet exhibit temperature dependent migration along the United States eastern seaboard [33–36]. Water temperature thus provides a good proxy for baitfish/prey availability. These baitfish form large spawning aggregations in coastal river mouths at successively lower latitudes along the United States eastern seaboard from October to January and at successively higher latitudes from February to May [33]. The timing of their presence corresponds with the southward and northward migration of the sharks and suggests that the sharks are following their food. So, whereas the shark migration correlates with temperature, prey abundance might be the causal link between the two factors.

The baitfish schools become largely depleted by midway through the blacktip sharks’ three month winter residency. The sharks almost certainly supplement their baitfish diet by foraging on the local fish population. The annual influx of a large number of upper trophic level predators likely creates an acute impact on the resident fish population, which could result in cascading effects through multiple trophic levels [37–39].

The blacktip shark abundance did not correlate with photoperiod. However, the onset of their departure from their overwintering habitat corresponds closely with the vernal equinox. Day length increases the most rapidly at the equinox and sharks might be using this rapid change in photoperiod as a cue to begin their northward migration. Photoperiod provides a more consistent temporal cue than changes in water temperature, which can vary from year to year. Photoperiod and water temperature have been correlated with shark movements [20–22] and the blacktips likely rely on both environmental factors to stimulate the onset of their northward migration.

Blacktip sharks are found year round in southeast Florida, albeit at much lower numbers outside of the peak winter season (Fig 4). They are reported throughout the year in the Florida Keys with a seasonal peak in abundance in late October to early November [16]. Their presence year round indicates that the summer warm water temperature does not impose a physiological limit to their distribution. These non-migratory individuals might be non-mating females in a resting year [16], or a subpopulation with a broader thermal tolerance. They might also represent individuals who shelter in cooler, deeper water and make only occasional forays to the nearshore environment where they were detected during the aerial survey flights.

### Aerial surveys

Aerial surveys are often used to quantify abundance of air breathing marine organisms, such as seabirds [40], turtles [41–43], and marine mammals [44–46], which necessarily come to the surface where they can be easily seen. Aerial surveys for sharks are typically conducted only for whale sharks [47–52] and basking sharks [53, 54]. The combination of their large size and surface association facilitates their visualization from the air. Smaller sharks have also been spotted in aerial surveys, either specifically targeting them [55, 56], or incidentally during marine mammal surveys [57]. Occasionally, massive schools of elasmobranchs have been documented from aerial photographs [58, 59] and in some instances, aggregations have been correlated with the presence of prey [60, 61].

Aerial surveys provide numerous advantages over conventional fishing surveys. For marine organisms, aerial surveys are non-disruptive; the animals are unaware of the survey vehicle and hence behaviors remain unaffected. In addition, aerial surveys are also non-selective and allow all animals in the area to be counted, including non-target species. Aerial surveys are efficient and cost effective permitting a large area of coastline to be sampled quickly. Finally, aerial surveys can provide abundance and density data that, along with other data sources (e.g. tagging and tracking studies), can help to identify critical habitats.

Although aerial surveys provide a number of attractive features, they are possible only under certain environmental conditions. For example, poor water clarity reduces the probability of detecting the target species. This is less of a problem with large organisms, such as whale sharks and basking sharks, whose size makes them relatively easy to see. The background against which the study organism is viewed also affects detectability. It is easier to spot a shark against a uniform background than against a patchwork mosaic such as a reef. Calm conditions with no surface waves contribute to water clarity and provide minimal distortion which facilitates identification of submerged organisms. Therefore, the ideal conditions for visualizing marine organisms require calm, clear water with a uniform background which contrasts with the dorsal coloration of the animal.

Even under ideal conditions, the morphological similarity of various shark species makes it impossible to distinguish species from the air, with a few exceptions. During survey flights, great hammerhead sharks (*Sphyrna mokarran*) could be identified by their head morphology and tiger sharks (*Galeocerdo cuvier*) could be identified by their blunt snout and much larger size compared to the blacktips. Anecdotally, the sharks in these aggregations are reported as blacktip or spinner sharks (*Carcharhinus brevipinna*). These two species appear very similar and close examination is necessary to distinguish them [1, 62]. Although spinner sharks are present in southeast Florida, the adults are found in deeper offshore waters and not immediately adjacent to the beach, as seen with the blacktips [2]. Beachgoers often see sharks jumping and spinning and conclude that the aggregations are composed of spinner sharks. However, the jumping and spinning behavior is common to both blacktip and spinner sharks [1, 16]. Longline fishing surveys conducted amongst the aggregating sharks confirm that the aggregations are composed almost exclusively of blacktip sharks (Kajiura unpublished).

The peak abundance was largely similar in 2011, 2012, and 2014 but with only about half the peak number of sharks counted in 2013 (Fig 4). The much lower number of sharks counted in 2013 is likely attributable to the reduced visibility that year due to beach renourishment projects along the survey transect. In that process, sand is pumped from offshore onto the beach to increase the width of the beach. This creates expansive, high turbidity conditions adjacent to the shore that make it impossible to view anything under the surface of the water. Sharks might have been present and not counted, or might have avoided the turbid conditions by moving farther offshore and outside the field of view of the survey transect.

Shark aggregations were often seen on the seaward side of the plane as well, but those sharks were outside the field of view of the survey transect and thus were not counted. As a result, the number of sharks directly counted in the survey provides an index of relative shark abundance and is not a population census. The sharks seen on the seaward side of the plane were still in fairly shallow water but sharks occurring at greater depths would be undetectable to an aerial survey. Therefore, the number of sharks directly counted is an underestimate of the total population and might represent only the tip of the iceberg of a much larger aggregation.

### Conservation

Shark populations continue to decline worldwide [63], including in the western Atlantic [64]. The abundance of upper trophic level predators is a critical metric of the health of an ecosystem [65] so the baseline data on shark abundance collected now can serve as a valuable benchmark for future studies [66]. The repeatability of the abundance estimates over several years suggests that monitoring the aggregation could provide an indicator of population size and perhaps management effectiveness. This is especially important given the variety of factors that could impact shark populations, including overfishing [63, 67], and ocean acidification, deoxygenation, and warming [68].

The large, densely packed blacktip aggregations present a potential management concern for these vulnerable K-selected species. The blacktip aggregation is highly predictable in space and time, which makes it especially vulnerable to exploitation. In Florida, spotter aircraft are used to direct gillnet fishermen to large aggregations [15]. Fishing regulations currently restrict harvest to one shark per person per day, or two sharks per vessel per day, in Florida state waters. These regulations protect the blacktip aggregations from exploitation within state waters, less than three nautical miles from shore [14]. In federal waters (>3 nautical miles offshore) blacktip sharks are able to be commercially harvested at a rate of 45 individuals per vessel per trip, with no limit to the number of trips per day [69]. Father north of Palm Beach County the shelf widens and the sharks have the potential to extend into federal waters, although the aggregations would likely be less condensed.

Marine organisms have been documented to occur at increasingly higher latitudes in response to warming oceans [70, 71]. Because the blacktip shark migration is closely correlated with water temperature, with very few sharks found when water temperatures exceed 25°C, warming oceans may shift the spatial range of future migrations to higher latitudes [72, 73]. As a result, southeast Florida may no longer represent the low latitude terminus of their migration. The resultant loss of this large annual influx of upper trophic level predators has the potential to create significant ecological ramifications, including cascading effects through multiple trophic levels [37–39].

To our knowledge, this blacktip shark migration is the single most massive seasonal shark migration seen in the western Atlantic. The compelling visual imagery of thousands of sharks immediately offshore captivates the public’s attention. It is possible to use this engagement to inform the public about the impact of overfishing, ocean acidification, and global ocean warming on local ecosystems and to promote conservation for these important marine predators.

## Supporting Information

S1 Movie Sample video clip from an aerial survey flight. This video was recorded south of the Palm Beach inlet. Thousands of sharks can be seen close to shore in this relatively short clip.
(MP4)

S1 Table Environmental parameters and shark abundance. Day of the year, water temperature, barometric pressure, photoperiod, number of sharks counted, and water clarity during survey flights for the study period.
(XLSX)
